# Indicators of insulin resistance as predictors of 28-day mortality in patients with VA-ECMO: a retrospective study

**DOI:** 10.3389/fmed.2025.1559780

**Published:** 2025-05-22

**Authors:** You Zhou, Zhi Cheng, Pingping Gu, Yu Zhang, Wanying Xu, Xin Wang

**Affiliations:** ^1^Department of Critical Care Medicine, The Second Affiliated Hospital of Nanjing Medical University, Nanjing, China; ^2^Binhai County People’s Hospital, Yancheng, China; ^3^Department of Geriatric Medicine, Suzhou Ninth People’s Hospital, Su Zhou, China

**Keywords:** insulin resistance, predictors, VA-ECMO, mortality, nomogram

## Abstract

**Background:**

Insulin resistance is closely related to adverse outcomes in critical illness, but its predictive value in patients with cardiogenic shock receiving venous-arterial extracorporeal membrane oxygenation (VA-ECMO) remains unclear.

**Methods:**

Patients with cardiogenic shock who received VA-ECMO treatment were retrospectively included. To evaluate the associations of insulin resistance indicators such as triglyceride-glucose (TyG), metabolic score for insulin resistance (METS-IR), triglyceride-to-high-density-lipoprotein cholesterol ratio (TG/HDL-C), and triglyceride glucose-body mass index (TyG-BMI) with 28-day mortality. A multi-stage modeling strategy was adopted. Firstly, risk factors were screened through univariate and multivariate Cox regression; Further combine Least Absolute Shrinkage and Selection Operator (LASSO) regression (L1 regularization), random forest and gradient boosting machine (GBM) for multi-method feature screening, and use ridge regression (L2 regularization) to control collinearity to construct a joint prediction model; Finally, the model efficacy was verified through C-index, time-dependent receiver operating characteristic (ROC) curve, calibration curve, decision curve analysis (DCA), net reclassification improvement index (NRI), and comprehensive discriminant improvement index (IDI).

**Results:**

TyG, METS-IR, TG/HDL-C, and TyG-BMI independently predicted an increased risk of death (all *p* < 0.01). Five core predictors were determined through multi-method screening: Sequential Organ Failure Assessment (SOFA) score, TyG, TG/HDL-C, hypertension, and diabetes. The joint model performed excellently in both the training set and the validation set (Training set: area under curve (AUC) = 0.923, C index = 0.847, NRI = 0.699, IDI = 0.175); Validation set: AUC = 0.901, C index = 0.846, NRI = 0.574, IDI = 0.148), and the DCA and calibration curve show its good efficacy.

**Conclusion:**

Insulin resistance indicators (TyG, TG/HDL-C) can independently and gradually predict the risk of death in patients with VA-ECMO. The model combined with indicators such as SOFA score has high discriminative power and clinical practicability. This provides new evidence for risk stratification based on the integration of metabolism and organ function, supporting the research exploration of targeted intervention for insulin resistance to improve prognosis.

## 1 Introduction

Insulin resistance is a critical contributor to the development of various metabolic disorders, including type 2 diabetes mellitus, cardiovascular disease, and chronic kidney disease (1–3). In recent years, the evaluation of insulin resistance has garnered significant attention, especially in critically ill patients, where it can significantly influence outcomes such as mortality and morbidity (4, 5). Venous-arterial extracorporeal membrane oxygenation (VA-ECMO), a life-saving intervention for patients with severe cardiopulmonary failure, presents an opportunity to explore the impact of insulin resistance on patient outcomes. Understanding this relationship is crucial for refining prognosis assessment and optimizing treatment strategies for patients on VA-ECMO.

Recent research has identified several biomarkers and indices to assess insulin resistance, including the triglyceride-glucose (TyG) index, metabolic score for insulin resistance (METS-IR), triglyceride-to-high-density lipoprotein cholesterol (TG/HDL-C) ratio, and triglyceride glucose-body mass (TyG-BMI) index. The TyG index, calculated as the natural logarithm of the product of triglyceride (TG) and glucose levels divided by two, has gained prominence as a simple and effective surrogate marker for insulin resistance (6). Similarly, METS-IR (7) and TyG-BMI (8) integrate metabolic parameters and provide a more comprehensive evaluation of insulin resistance. The TG/HDL-C ratio, a novel biomarker for metabolic syndrome, has been shown to correlate significantly with cardiovascular disease risk in the general population (9).

Although progress has been made in understanding insulin resistance, its specific impact on patients receiving VA-ECMO has not been fully explored, especially in patients with cardiogenic shock. Managing critically ill patients requires an in-depth understanding of how metabolic factors, such as insulin resistance, interact with clinical conditions and therapeutic interventions. Insulin resistance is known to elevate inflammatory markers, potentially worsening acute disease severity and adversely affecting the prognosis of patients on VA-ECMO (10). Furthermore, comorbidities such as hypertension, diabetes, and other metabolic disorders, which are closely associated with insulin resistance, can complicate patient outcomes and exacerbate prognostic challenges (11).

This retrospective study aimed to investigate the relationship between insulin resistance indicators and the 28-day mortality rate in patients treated with VA-ECMO. These assessments help elucidate the role of insulin resistance in influencing outcomes for this high-risk population. The study findings expand current knowledge and may guide future management strategies to improve survival rates. A deeper understanding of metabolic dysregulation in critically ill patients is essential for optimizing care and enhancing outcomes in the complex setting of VA-ECMO treatment.

## 2 Materials and methods

### 2.1 Study population

From January 2023 to March 2025, a total of 268 patients who received VA-ECMO treatment in the intensive care unit and emergency intensive care unit were collected in Binhai County People’s Hospital and the Second Affiliated People’s Hospital of Nanjing Medical University. The study was approved and supervised by the Ethics Committee of Binhai County People’s Hospital (Approval number: 2024-BYKYLL-015) and adhered to the principles outlined in the Declaration of Helsinki (revised in 2013). Informed consent was obtained from all participants or their legally authorized representatives before inclusion in the study. The inclusion criteria were: (1) patients aged ≥ 18 years; (2) patients treated with VA-ECMO for cardiogenic shock (such as acute myocardial infarction, fulminant myocarditis, acute decompensation of end-stage heart failure, etc.); (3) VA-ECMO treatment lasted for more than 24 h; (4) patients who signed informed consent. Exclusion criteria included: (1) patients with incomplete laboratory indicators; (2) the presence of malignant tumors; (3) severe sepsis or septic shock; (4) long-term (> 7 days) use of glucocorticoids or other immunomodulators before admission; (5) history of metabolic diseases (such as thyroid dysfunction, chronic liver and kidney diseases causing metabolic disorders); (6) Those who died or were weaned within 24 h after emergency VA-ECMO implantation (to avoid interference with analysis by extremely short-term cases); (7) refusal to provide informed consent.

### 2.2 Clinical data collection and follow-up

The study collected the following variables: (1) basic information: age, sex, body mass index (BMI), and waist circumference (WC); (2) laboratory indicators: white blood cells (WBC), platelets (PLT), lactic acid (Lac), cholesterol, albumin, TG, high-density lipoprotein cholesterol (HDL-C), glutamic oxaloacetic transaminase (AST), glutamic pyruvic transaminase (ALT), creatinine (CR), urea nitrogen (BUN), fasting blood glucose (FBG), interleukin-6 (IL-6) and C-reactive protein (CRP); (3) underlying conditions: hypertension, diabetes, and acute kidney injury (AKI); (4) disease severity scores: sequential organ failure assessment (SOFA) and acute physiology and chronic health evaluation II (APACHE II). All laboratory data and severity scores were collected by trained professionals using standardized procedures and automated systems to ensure consistency. Clinical examiners conducted the assessments, and specialists performed the scoring. Information on pre-existing conditions was obtained primarily from patient’s medical histories. Follow-up was conducted through outpatient visits or telephone interviews, with survival data collected at 28 days post-VA-ECMO treatment.

### 2.3 Calculation of insulin resistance indices

The following formulas were used to calculate insulin resistance indices: TyG index (12) = Ln[TG (mg/dL) × blood glucose (mg/dL)/2]; METS-IR (13) = Ln[2 × blood glucose (mg/dL) +TG (mg/dL)] × BMI/Ln HDL-C (mg/dL); TG/HDL-C (14) = TG (mg/dL) /HDL-C (mg/dL); TyG-BMI index = TyG × BMI.

### 2.4 Statistical analysis

The baseline characteristics of all included patients were stratified based on whether they survived 28 days after ECMO treatment. The four indicators of TyG, METS_IR, TG/HDL_C, and TyG_BMI were grouped according to quartiles and divided into Q1, Q2, Q3, and Q4. Variables that do not conform to the normal distribution are expressed as interquartile ranges and compared using the Wilcoxon rank sum test. Categorical variables were expressed as percentages and compared using the chi-square test. To obtain the relationship between the target variable and the patient outcome, in the survival analysis, the Kaplan-Meier (K-M) curve was used to illustrate the survival trends within 28 days in patients with VA-ECMO in different groups of TyG, METS_IR, TG/HDL_C, and TyG_BMI. Subsequently, we conducted a univariate Cox regression analysis to further clarify the relationship between the levels of TyG, METS_IR, TG/HDL_C, and TyG_BMI and the 28-day all-cause mortality of patients with VA-ECMO. To further explain the potential confounding factors in the Cox regression model, we conducted a multivariate Cox regression analysis and adjusted for various covariates to construct three models. Restrictive cube plots (RCS) and threshold effect analysis were used to determine potential inflection points in order to evaluate the linear or nonlinear relationships between clinical outcomes and the levels of TyG, METS_IR, TG/HDL_C, and TyG_BMI as continuous variables. To identify the predictor variables most relevant to the target outcome and construct a robust diagnostic model, this study adopts a multi-stage feature screening and validation strategy. The specific steps are as follows: (1) Multi-method joint variable initial screening and machine learning feature selection were used to quantify the contribution of candidate variables to the outcome through three methods: Lasso regression (L1 regularization), random forest (based on Gini impurity), and gradient boosting machine (GBM). Each method retained the top 6 variables in terms of contribution. (2) Cross-validation and variable integration: Extract the variables (intersections) jointly screened out by the three methods, or the variables selected simultaneously by at least two methods, as well as clinical experience, to form an initial screening variable pool, thereby enhancing the robustness of feature selection. (3) Collinearity control and final variable determination: Conduct hill regression analysis (L2 regularization) on the initial screening variable pool. Constrain the coefficients of highly correlated variables through penalty terms to reduce the impact of multicollinearity on model stability, and screen the variables finally included in the model based on the significance of the regression coefficients. (4) Nomogram construction and model validation: Clinical predictive nomograms were constructed using the determined variables mentioned above, and their performance was evaluated through the following multi-dimensional index system: Discriminative ability: Receiver operating characteristic curve (ROC) and area under the curve (AUC); Classification improvement degree: Net weight classification improvement index (NRI) and comprehensive discriminant improvement index (IDI); Clinical practicality: Decision Curve analysis (DCA) quantifies the clinical net benefits of the model under different threshold probabilities; Calibration degree: The Calibration Curve combined with the Hosmer-Lemeshow test assesses the consistency between the predicted probability and the actual risk. All statistical analyses were performed using R software (version 4.3.0) and STATA 17.0 (64-bit), with bilateral *p*-values < 0.05 considered statistically significant.

## 3 Results

### 3.1 Demographic and clinical characteristics of patients on VA-ECMO

A total of 268 patients were included in the analysis in this study. The baseline characteristics of the study population are shown in Table 1. Among the patients treated with VA-ECMO, 141 cases (52.62%) died within 28 days after treatment, and 127 cases (47.38%) survived. Compared with the survivors, the patients who died were older, and the levels of WBC, CRP, ALT, Lac, Cholesterol, TG, FBG, SOFA score and APACHE II score were significantly increased, while the levels of PLT, albumin and HDL_C were significantly lower. Deceased patients are prone to complications such as hypertension and diabetes. For the analysis of the levels of the target variables TyG, METS_IR, TG/HDL_C and TyG_BMI, it was found that the levels of patients who died were significantly higher. The above results all had statistically significant differences (all *p*-value < 0.05).

**TABLE 1:** Demographic and clinical characteristics of patients treated with VA-ECMO.

Characteristic	Total no. (%)	Survival	Dead	*P-*value
		**No. (%)**	**No. (%)**	
Total	268	127 (47.38)	141 (52.62)	
Age, (years)	60.50 (51.75, 69.00)	59.00 (45.00, 67.50)	63.00 (55.00, 70.00)	**0.007**
Sex, *n* (%)				0.877
Female	100 (37.31)	48 (37.80)	52 (36.88)	
Male	168 (62.69)	79 (62.20)	89 (63.12)	
BMI (kg/m^2^)	24.22 (22.31, 26.20)	23.73 (21.97, 25.95)	24.45 (22.83, 26.56)	0.238
WC (cm)	78.50 (74.00, 85.00)	78.00 (74.00, 84.00)	80.00 (73.00, 86.00)	0.371
WBC (10^9^/L)	13.53 (9.55, 18.45)	12.87 (9.02, 17.25)	13.90 (10.73, 19.76)	**0.046**
PLT (10^9^/L)	127.00 (81.00, 187.75)	163.00 (111.50, 215.00)	102.00 (68.00, 158.00)	**<0.001**
AST (U/L)	38.50 (22.87, 130.00)	35.70 (22.75, 125.30)	42.00 (23.50, 130.00)	0.313
ALT (U/L)	58.00 (37.15, 192.28)	48.60 (33.00, 114.00)	83.00 (39.80, 318.50)	**0.001**
Albumin (g/l)	31.30 (28.25, 35.00)	33.00 (29.35, 35.80)	30.00 (27.70, 34.00)	**<0.001**
CR (mg/dL)	105.30 (78.60, 150.07)	99.00 (75.95, 141.44)	108.00 (80.20, 159.12)	0.182
BUN (mg/dL)	8.20 (6.43, 11.75)	8.10 (6.20, 11.78)	8.21 (6.60, 11.26)	0.527
Cholesterol (mmol/l)	3.84 (3.48, 4.30)	3.73 (3.26, 4.12)	4.11 (3.60, 4.58)	**<0.001**
Lac (mmol/L)	2.95 (1.58, 9.65)	2.50 (1.50, 6.65)	4.70 (1.60, 13.30)	**0.022**
TG (mg/dl)	159.48 (123.81, 211.92)	132.90 (104.02, 161.14)	196.19 (156.80, 241.76)	**<0.001**
HDL_C (mg/dl)	46.72 (40.44, 54.92)	55.21 (43.82, 60.62)	43.24 (39.38, 46.72)	**<0.001**
FBG (mg/dL)	217.72 (150.97, 294.45)	162.56 (120.64, 216.00)	270.60 (216.30, 324.60)	**<0.001**
IL_6 (pg/mL)	268.15 (182.55, 416.65)	266.37 (156.75, 454.15)	269.54 (211.90, 387.20)	0.507
CRP (mg/L)	59.41 (26.34, 106.18)	53.87 (20.32, 88.59)	70.92 (36.80, 111.00)	**0.004**
TyG	9.81 (9.14, 10.26)	9.25 (8.86, 9.72)	10.15 (9.82, 10.51)	**<0.001**
METS IR	40.61 (36.21, 44.19)	37.64 (34.59, 41.89)	42.75 (39.43, 47.07)	**<0.001**
TG/HDL_C	3.40 (2.50, 4.92)	2.53 (2.05, 3.00)	4.59 (3.56, 5.66)	**<0.001**
TyG_BMI	235.19 (212.29, 261.31)	224.89 (199.30, 241.50)	244.88 (226.75, 272.06)	**<0.001**
SOFA	9.00 (8.00, 13.00)	8.00 (6.50, 9.00)	12.00 (9.00, 15.00)	**<0.001**
APACHE II	29.00 (27.00, 33.00)	28.00 (25.50, 33.00)	30.00 (29.00, 34.00)	**<0.001**
Hypertension, *n* (%)				**<0.001**
No	137 (51.12)	82 (64.57)	55 (39.01)	
Yes	131 (48.88)	45 (35.43)	86 (60.99)	
Diabetes, *n* (%)				**<0.001**
No	166 (61.94)	94 (74.02)	72 (51.06)	
Yes	102 (38.06)	33 (25.98)	69 (48.94)	
AKI, *n* (%)				0.101
No	157 (58.58)	81 (63.78)	76 (53.90)	
Yes	111 (41.42)	46 (36.22)	65 (46.10)	
TyG, *n* (%)				**<0.001**
Q1	54 (20.15)	47 (37.01)	7 (4.96)	
Q2	79 (29.48)	52 (40.94)	27 (19.15)	
Q3	71 (26.49)	24 (18.90)	47 (33.33)	
Q4	64 (23.88)	4 (3.15)	60 (42.55)	
METS_IR, *n* (%)				**<0.001**
Q1	66 (24.63)	48 (37.80)	18 (12.77)	
Q2	67 (25.00)	38 (29.92)	29 (20.57)	
Q3	68 (25.37)	28 (22.05)	40 (28.37)	
Q4	67 (25.00)	13 (10.24)	54 (38.30)	
TG/HDL_C, *n* (%)				**<0.001**
Q1	68 (25.37)	61 (48.03)	7 (4.96)	
Q2	66 (24.63)	45 (35.43)	21 (14.89)	
Q3	64 (23.88)	10 (7.87)	54 (38.30)	
Q4	70 (26.12)	11 (8.66)	59 (41.84)	
TyG_ BMI, *n* (%)				**<0.001**
Q1	67 (25.00)	48 (37.80)	19 (13.48)	
Q2	66 (24.63)	36 (28.35)	30 (21.28)	
Q3	64 (23.88)	21 (16.54)	43 (30.50)	
Q4	71 (26.49)	22 (17.32)	49 (34.75)	

Bold *p*-values <0.05 considered statistically significant.

### 3.2 Prognostic factors for 28-day survival of patients after VA-ECMO treatment

**FIGURE 1:**
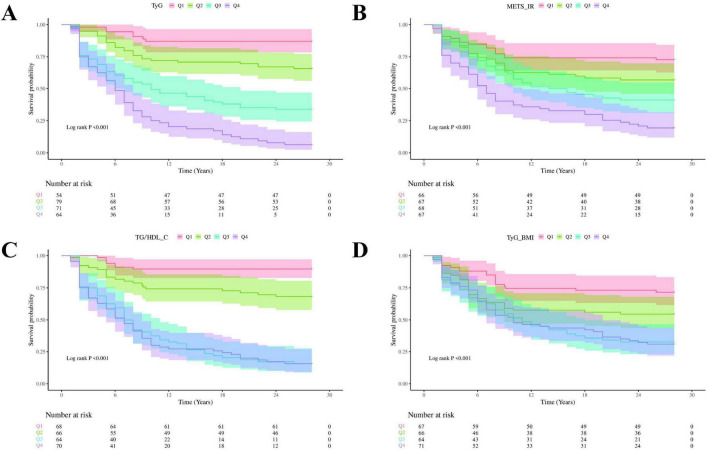
The 28-day survival Kaplan-Meier survival curve of patients after VA-ECMO treatment. **(A)** TyG; **(B)** METS_IR; **(C)** TG/HDL_C; **(D)** TyG_BMI.

Based on the target variables TyG, METS_IR, TG/HDL_C, and TyG_BMI, the relationship between the 28-day all-cause mortality of patients in different groups was compared. The study group plotted the Kaplan-Meier survival curve (Figure 1). Analysis of the TyG index revealed that the 28-day mortality rate was the highest in group Q4 and the survival rate was the highest in group Q1, with significant differences (log-rank *P* < 0.001) (Figure 1A). The METS_IR index was analyzed, and the results were consistent with those of the TyG group. The 28-day mortality risk from low to high was Q1, Q2, Q3, and Q4 respectively, with significant differences (log-rank *P* < 0.001) (Figure 1B). The TG/HDL_C and TyG_BMI indicators were analyzed. It was found that although the results were consistent with the trend of the above indicators, there was a crossover for patients in the Q3 and Q4 groups in the later stage, and there were significant differences between the two groups of indicators (log-rank *P* < 0.001) (Figures 1C,D).

There are significant differences between the two outcomes among different levels of TyG, METS_IR, TG/HDL_C, and TyG_BMI. For further survival analysis, the research group conducted a univariate Cox regression analysis (Table 2). Age, Cholesterol, Lac, TG, FBG, CRP, SOFA, Diabetes, and Hypertension are all risk factors. The higher the index level is, the higher the risk of death in patients with VA-ECMO is, and the difference is statistically significant (all *p*-value < 0.05). PLT, Albumin and HDL_C were protective factors, and there were statistically significant differences (all *p*-value < 0.05). The levels of TyG, METS_IR, TG/HDL_C, and TyG_BMI were risk factors, and there were statistically significant differences (all *p*-value < 0.05).

**TABLE 2:** Univariate analysis of 28-day all-cause mortality in VA-ECMO patients.

Characteristic	Hazard ratio (95% CI)	*P*-value
**Gender, *n* (%)**		
Female	Reference	
Male	1.02 (0.73,1.44)	0.897
Age (years)	**1.02 (1.01,1.03)**	**0.009**
BMI (kg/m^2^)	1.00 (0.95,1.04)	0.914
WC (cm)	1.00 (0.99,1.02)	0.650
WBC (10^9^/L)	1.02 (1.00,1.04)	0.081
PLT (10^9^/L)	**0.94 (0.90,0.98)**	**<0.001**
AST (U/L)	1.00 (1.00,1.00)	0.393
ALT (U/L)	1.00 (1.00,1.00)	0.242
Albumin (g/l)	**0.94 (0.90,0.97)**	**<0.001**
CR (mg/dL)	1.00 (1.00,1.00)	0.419
BUN (mg/dL)	0.98 (0.95,1.01)	0.222
Cholesterol (mmol/l)	**1.30 (1.12,1.51)**	**<0.001**
Lac (mmol/L)	**1.04 (1.01,1.06)**	**<0.001**
TG (mg/dl)	**1.02 (1.01,1.04)**	**<0.001**
HDL_C (mg/dl)	**0.96 (0.95,0.97)**	**<0.001**
FBG (mg/dL)	**1.03 (1.01,1.06)**	**<0.001**
IL_6 (pg/mL)	1.00 (1.00,1.00)	0.371
CRP (mg/L)	**1.02 (1.01,1.05)**	**0.013**
SOFA	**1.39 (1.31,1.47)**	**<0.001**
APACHE II	1.00 (0.99,1.02)	0.636
TyG	**3.39 (2.62,4.38)**	**<0.001**
METS_IR	**1.06 (1.04,1.09)**	**<0.001**
TG/HDL_C	**1.18 (1.13,1.24)**	**<0.001**
TyG_BMI	**1.02 (1.01,1.04)**	**<0.001**
**TyG**		
Q1	Reference	
Q2	**2.98 (1.30,6.84)**	**0.010**
Q3	**4.79 (2.52,7.26)**	**<0.001**
Q4	**5.80 (3.72,9.60)**	**<0.001**
**METS_IR**		
Q1	Reference	
Q2	1.76 (0.98,3.18)	0.059
Q3	**2.55 (1.46,4.45)**	**0.001**
Q4	**4.43 (2.59,7.57)**	**<0.001**
**TG/HDL_C**		
Q1	Reference	
Q2	**3.52 (1.50,5.28)**	**0.004**
Q3	**4.63 (2.63,7.31)**	**<0.001**
Q4	**5.46 (3.03,9.39)**	**<0.001**
**TyG_BMI**		
Q1	Reference	
Q2	**1.89 (1.06,3.35)**	**0.030**
Q3	**3.10 (1.80,5.32)**	**<0.001**
Q4	**3.16 (1.86,5.38)**	**<0.001**
**Hypertension**		
No	Reference	
Yes	**1.88 (1.34,2.64)**	**<0.001**
**Diabetes**		
No	Reference	
Yes	**1.87 (1.34,2.61)**	**<0.001**
**AKI**		
No	Reference	
Yes	1.30 (0.94,1.81)	0.118

Bold *p*-values <0.05 considered statistically significant.

To consider the factors of different covariates and determine whether the target variables TyG, METS_IR, TG/HDL_C, and TyG_BMI were independent factors for patient outcomes, we constructed three multivariate Cox regressions (Table 3) and calculated the variance inflation factor (VIF) (Supplementary Table S1). The VIF of each group of factors was calculated and it was found that they were all lower than five, suggesting that the collinearity among each index was relatively low. The TyG index was analyzed. In the Cox proportional hazards model 1 without adjusting for any variables, the ungrouped TyG level was significantly associated with the 28-day all-cause mortality rate of VA-ECMO patients (HR = 3.4, 95% CI 2.6–4.4; *P* < 0.001); Taking group Q1 as the reference, the risk of death in the other three groups of patients was significantly increased, among which the risk in group Q4 was the highest (HR = 5.8, 95% CI 3.7–9.6; *P* < 0.001); After adjusting for some confounding factor models 2, the results were consistent with those of Model 1. After adjusting for the complete variable model 3, the results were basically consistent with those of the previous two models. The risk degree of death at the ungrouped TyG level was reduced compared with before (HR = 1.8, 95% CI 1.2–2.5; *P* = 0.001). The same was true for the grouped TyG patients, among which the risk in group Q4 remained the highest (HR = 4.7, 95% CI 2.3–8.4; *P* = 0.002). Based on the above results, it was found that a high level of TyG is a risk factor for the death of patients. Analyze the three models of METS_IR. For the METS_IR of continuous variables, the higher the level, the greater the risk of death. For the classified METS_IR, patients in group Q4 had the highest risk, and the risk in group Q3 was higher than that in group Q2. However, although patients in group Q2 were risk factors, there was no statistically significant difference. The TG/HDL_C index was analyzed. Model 2 and Model 3 found that patients in group Q3 had the highest risk (Model 2: HR = 9.4, 95% CI 4.1–21.4; *P* < 0.001); Model 3: HR = 9.5, 95% CI 4.1–22.5; *P* < 0.001). The TyG_BMI index was analyzed. Only the result of model 1 had a statistically significant difference, and it was also a risk factor. Group Q4 had the highest risk (HR = 3.2, 95% CI 1.9–5.4; *P* < 0.001). Through multivariate cox regression analysis, we found that some subgroups of TyG, METS_IR, TG/HDL_C, and TyG_BMI were significantly associated with the 28-day mortality risk of patients with VA-ECMO and were risk factors.

**TABLE 3:** Multivariable cox regression analysis of TyG, METS_IR, TG/HDL_C, and TyG_BMI with the 28-day all-cause mortality rate in ECMO patients.

Outcomes exposure	Model 1 HR (95% CI, *P*)	Model 2 HR (95% CI, *P*)	Model 3 HR (95% CI, *P*)
TyG	**3.4 (2.6, 4.4) < 0.001**	**2.2 (1.6, 3.0) < 0.001**	**1.8 (1.2, 2.5) 0.001**
**TyG group**			
Q1	Ref	Ref	Ref
Q2	**3.0 (1.3,6.8) 0.010**	2.7 (0.9, 5.5) 0.082	**2.4 (1.1, 5.3) 0.037**
Q3	**4.8 (2.5,7.9) < 0.001**	**4.4 (1.8, 7.6) 0.001**	**4.2 (1.6, 7.1) 0.001**
Q4	**5.8 (3.7,9.6) < 0.001**	**5.2 (2.5, 8.8) < 0.001**	**4.7 (2.3, 8.4) 0.001**
METS_IR	**1.1 (1.0,1.1) < 0.001**	**1.0 (1.0, 1.1) 0.003**	**1.1 (1.0, 1.1) 0.002**
**METS_IR group**			
Q1	Ref	Ref	Ref
Q2	1.8 (1.0, 3.2) 0.059	1.5 (0.8, 2.7) 0.239	1.4 (0.8, 2.8) 0.264
Q3	**2.5 (1.5, 4.4) 0.001**	**1.9 (1.0, 3.6) 0.037**	1.6 (0.8, 3.1) 0.168
Q4	**4.4 (2.6,7.6) < 0.001**	**2.3 (1.2, 4.2) 0.009**	**2.1 (1.1, 3.9) 0.018**
**TG/HDL_C**			
TG/HDL_C group	**1.2 (1.0,1.2) < 0.001**	**1.1 (1.0, 1.2) 0.001**	**1.1 (1.0, 1.2) 0.039**
Q1	Ref	Ref	Ref
Q2	**3.5 (1.5,5.3) 0.004**	**3.0 (1.2, 7.1) 0.015**	**3.0 (1.3, 7.4) 0.014**
Q3	**4.6 (2.6,7.3) < 0.001**	**9.4 (4.1, 21.4) < 0.001**	**9.5 (4.1, 22.1) < 0.001**
Q4	**5.5 (3.0,9.4) < 0.001**	**8.7 (3.9, 19.8) < 0.001**	**8.0 (3.4, 18.8) < 0.001**
TyG_BMI	**1.1 (1.0,1.1) < 0.001**	1.0 (1.0, 1.0) 0.114	1.0 (1.0, 1.0) 0.081
**TyG_BMI group**			
Q1	Ref	Ref	Ref
Q2	**1.9 (1.1, 3.4) 0.030**	1.4 (0.8, 2.5) 0.293	1.3 (0.7, 2.5) 0.417
Q3	**3.1 (1.8, 5.3) < 0.001**	1.5 (0.8, 2.8) 0.163	1.4 (0.8, 2.6) 0.245
Q4	**3.2 (1.9, 5.4) < 0.001**	1.5 (0.8, 2.6) 0.207	1.3 (0.7, 2.4) 0.388

Bold represents statistically significant correlation with the outcome and *p*-value less than 0.05. Model 1: no covariates were adjusted. Model 2: adjusted for age, gender (male, female), Hypertension (yes, no), Diabetes (yes, no), acute kidney injury (yes, no), SOFA and APACHE II. Model 3: adjusted for Model 2 plus laboratory indicators.

### 3.3 Relationship between insulin resistance index and survival prognosis of patients on VA-ECMO

**FIGURE 2:**
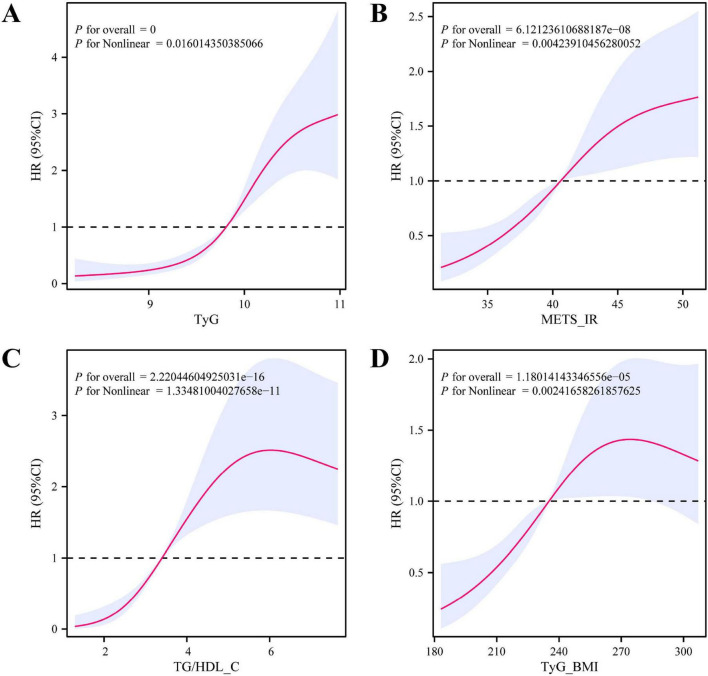
RCS plot of the relationship between the levels of TyG, METS_IR, TG/HDL_C, and TyG_BMI and the 28-day mortality rate of patients with VA-ECMO. **(A)** TyG; **(B)** METS_IR; **(C)** TG/HDL_C; **(D)** TyG_BMI.

To evaluate the relationship between the levels of indicators such as TyG, METS_IR, TG/HDL_C, and TyG_BMI and the 28-day mortality rate of patients with VA-ECMO more intuitively, the RCS plot was plotted (Figure 2). The levels of TyG, METS_IR, TG/HDL_C, and TyG_BMI were all positively correlated with the patient outcomes, but not completely linearly correlated (Figures 2A–D). Threshold effect analysis determined the key inflection points. First, for the TyG analysis, the inflection point was 10.04. When < 10.04: HR = 5.88 (3.00–11.53), *p* = 0.001; When ≥ 10.04: HR = 2.02 (1.07–3.80), *p* = 0.001. The logarithmic likelihood ratio test showed *p* = 0.061, suggesting that there was no obvious threshold effect point for the relationship between TyG and the outcome. The inflection point of METS_IR was 46.90, and there was a threshold effect in the association between METS_IR and the outcome (*P* for likelihood test = 0.003). Overall, METS_IR was positively correlated with the outcome, with HR = 1.06 (1.04–1.09). When METS_IR < 46.90, HR = 1.12 (1.07–1.18). When METS_IR ≥ 46.90, there was no statistical difference. The inflection point of TG/HDL_C was 3.92. There was a threshold effect of TG/HDL_C (*P* for likelihood test < 0.001), and TG/HDL_C was also positively correlated with the outcome, with HR = 1.18 (1.13–1.24). The inflection point of TyG_BMI was 246.58. TyG_BMI also had a threshold effect (*P* for likelihood test < 0.001). TyG_BMI was positively associated with the outcome, with HR = 1.01 (1.00–1.01). Based on the above results, the different risk changes and prediction trends of different indicators for the results were revealed. Among them, the target variable shows a relatively obvious positive trend, and the higher value is always associated with the increase of the prediction effect. Subsequently, we also analyzed other factors (Supplementary Figure S1). Factors such as Cholesterol, Lac, ALT, TG, FBG, CRP, and SOFA were positively correlated with patient outcomes, while factors such as PLT, Albumin, and HDL_C were negatively correlated with patient outcomes.

### 3.4 Critical factors for 28-day mortality in patients treated with VA-ECMO

**FIGURE 3:**
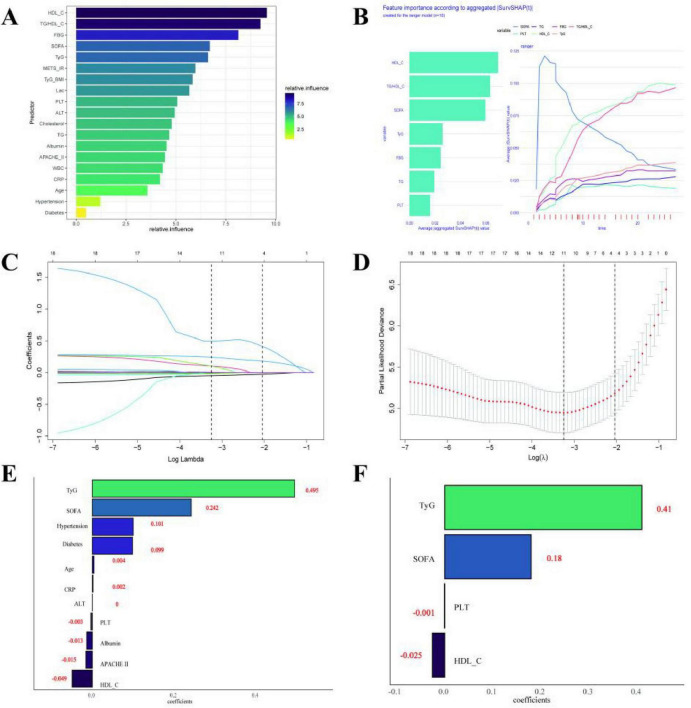
Determine the screening process of key factor variables and model construction for the 28-day mortality rate of patients treated with VA-ECMO. **(A)** GBM screened out HDL_C, TG/HDL_C, FBG, SOFA score, TyG index and METS_IR; **(B)** Random forest was used to screen out HDL_C, TG/HDL_C, SOFA score, TyG index, FBG and TG; **(C–F)** Lasso regression was used to screen out TyG index, SOFA score, hypertension, history of diabetes, HDL_C and APACHE II score.

Variable screening and model construction process: To identify the predictor variables most relevant to the target outcome and construct a robust diagnostic model, this study adopts a multi-stage feature screening and validation strategy. The specific steps are as follows: Multi-method joint variable initial screening, quantifying the contribution of candidate variables to the outcome through the following three machine learning methods, respectively (Figure 3) : GBM screened out HDL_C, TG/HDL_C, FBG, SOFA score, TyG index and METS_IR (Figure 3A); Random forest was used to screen out HDL_C, TG/HDL_C, SOFA score, TyG index, FBG and TG (Figure 3B); Lasso regression was used to screen out TyG index, SOFA score, hypertension, history of diabetes, HDL_C and APACHE II score (Figures 3C–F). Cross-integration and clinical validation were conducted to extract variables and clinical experiences that were jointly screened by at least two methods, including TyG index, TG/HDL_C, SOFA score, history of hypertension, and diabetes. Redundant variable elimination: Although HDL_C and FBG repeatedly occur in multiple methods, since they are respectively components of the TyG index (including FBG) and TG/HDL_C (including HDL_C), in order to avoid multicollinearity interference, they are excluded after discussion by the clinical expert group.

### 3.5 Collinearity control, model construction and evaluation

**FIGURE 4:**
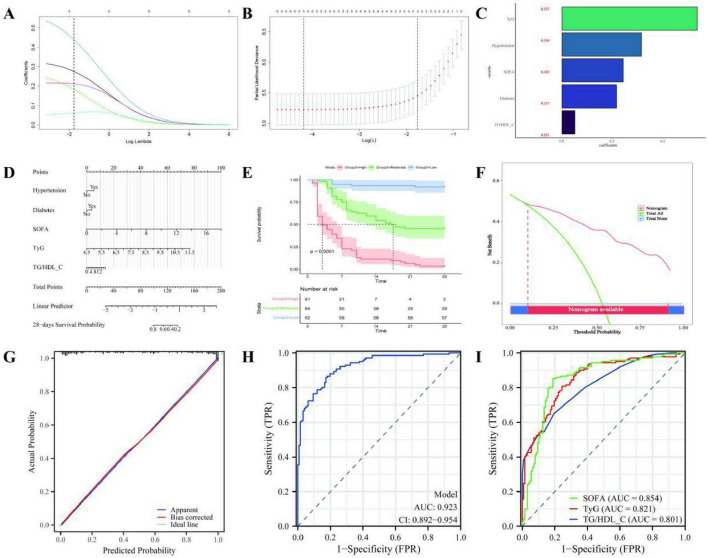
Collinearity control, model construction and evaluation**. (A–C)** The penalty term λ is determined through 10-fold cross-validation; **(D)** Construct the clinical prediction nomogram; **(E)** K-M survival analysis was conducted by stratifying low, medium and high risks based on individualized risk scores; **(F)** DCA; **(G)** Calibration degree; **(H)** Model efficacy validation and clinical applicability were conducted using a 7:3 ratio split dataset for internal and external validation, and comprehensive evaluation indicators were carried out; **(I)** The predictive efficacy of TyG index, TG/HDL-C, SOFA score and nomogram model for the 28-day survival outcome of patients with VA-ECMO was compared through ROC curve analysis.

To control multicollinearity among variables and verify the predictive efficacy, the following analysis strategies were adopted in this study: collinearity test of variables and weight evaluation. The independence of the final screened variables (TyG index, TG/HDL_C, SOFA score, hypertension, and history of diabetes) was evaluated through ridge regression analysis (the penalty term λ was determined by 10-fold cross-validation, Figures 4A–C). The results show that all variables VIF < 3.0 (the threshold is set to 5); Prediction contribution ranking: Based on the standardized regression coefficient, the importance of the variables is in the order of TyG index (β = 0.537), hypertension (β = 0.316), SOFA score (β = 0.243), history of diabetes (β = 0.217), and TG/HDL_C (β = 0.051). Nomogram model construction and risk stratification: A clinical prediction nomogram was constructed based on the above variables (Figure 4D), the individualized risk score was calculated, and the quartiles of the risk score were used as the cut-off values (low risk: < 74.84); Medium-risk: 74.84–99.60 High-risk: > 99.60) Stratify. K-M survival analysis showed that the 28-day all-cause mortality rate in the high-risk group was significantly higher than that in the medium and low-risk groups (log-rank *p* < 0.001, Figure 4E). Model performance validation and clinical applicability: Internal and external validation was conducted using a 7:3 ratio split dataset. The comprehensive evaluation indicators are as follows: Discrimination: The AUC of the ROC of the training set and the validation set was 0.923 (95% CI: 0.892–0.954) and 0.901 (95% CI: 0.863–0.939), respectively, and the C-index was 0.847 and 0.846, respectively (Figure 4H); Reclassification efficiency: The NRI of the training set was 0.699 (95% CI: 0.473–0.951) and IDI was 0.175 (95% CI: 0.111–0.261), while the NRI of the validation set was 0.574 (95% CI: 0.396–0.752) and IDI was 0.148 (95% CI: 0.086–0.234); Calibration degree: The calibration curve approaches the

ideal diagonal (Brier score = 0.41, Figure 4G); Clinical utility: DCA shows that within a wide threshold probability range (10–90%) (Figure 4F).

### 3.6 Predictive value of insulin resistance index for 28-day survival prognosis

To further evaluate the prognostic discriminatory ability of the combined model compared with a single indicator, the predictive efficacy of TyG, TG/HDL-C, SOFA score, and nomogram model for the 28-day survival outcome of patients with VA-ECMO was compared through ROC curve analysis (Figure 4I). The results show: Single indicator: TyG (AUC = 0.821, 95% CI 0.776–0.866), TG/HDL-C (AUC = 0.801, 95% CI 0.751–0.851), SOFA score (AUC = 0.854, 95% CI 0.812–0.896); Combined model: The AUC of the nomogram model was significantly higher than that of all single indicators (AUC = 0.923, 95% CI: 0.892–0.954).

### 3.7 Hierarchical analysis

To further evaluate the predictive stability and population heterogeneity of the TyG and TG/HDL-C for the 28-day mortality risk of patients with VA-ECMO, this study conducted stratified analyses based on gender, age (< 65 years old vs. ≥ 65 years old), BMI (< 25 kg/m^2^ vs. ≥ 25 kg/m^2^), and comorbidity status (hypertension, diabetes, kidney injury) (Table 4). The key findings are as follows: In the subgroup analysis of TyG and outcomes, significant statistical differences were observed in the relationship between TyG and various subgroup factors. We observed that the TyG level in group Q4 was significantly associated with an increased risk of patient death. Specifically, this relationship was more significant in men under 65 years old, patients with BMI < 25 kg/m, those with hypertension, diabetes, and kidney injury than in the other group, and the risk level was higher. In the analysis of TG/HDL-C and outcomes, it was also observed that there were significant statistical differences in the relationship between TG/HDL-C and various subgroup factors. Analysis revealed that with group Q1 as a reference, the TG/HDL-C level in group Q4 was significantly associated with an increased risk of patient death. Specifically, this relationship was more significant in women, patients under 65 years old, patients with BMI ≥ 25 kg/m, those with hypertension or diabetes, and those without kidney injury compared with the other group of patients, and the risk level was higher.

**TABLE 4:** Subgroup analysis of different factors.

Subgroup	Variables	TG/HDL_C	*p*	TyG	*p*
		**HR (95% CI)**		**HR (95% CI)**	
**Sex**					
**Female**					
	Q1	Reference		Reference	
	Q2	4.34 (1.12, 9.82)	0.033	4.29 (0.81,8.75)	0.078
	Q3	5.78 (2.09, 11.80)	<0.001	5.84 (2.50,11.02)	0.004
	Q4	6.78 (2.85, 12.89)	<0.001	7.01 (4.94,15.27)	<0.001
**Male**					
	Q1	Reference		Reference	
	Q2	3.03 (0.99, 9.21)	0.051	2.45 (0.96,6.33)	0.058
	Q3	4.74 (2.14, 8.22)	<0.001	5.90 (2.45,12.19)	<0.001
	Q4	5.95 (2.63, 9.50)	<0.001	7.02 (4.61,16.31)	<0.001
**Age (years)**					
**<60**					
	Q1	Reference		Reference	
	Q2	5.01 (1.04, 24.12)	0.044	3.04 (0.68,13.59)	0.145
	Q3	6.55 (2.27, 12.40)	<0.001	5.69 (2.81,11.75)	0.006
	Q4	7.33 (3.00, 16.98)	<0.001	8.06 (4.03,17.23)	<0.001
**≥60**					
	Q1	Reference		Reference	
	Q2	2.72 (0.97, 7.54)	0.055	3.289 (1.19,9.05)	0.021
	Q3	4.65 (3.69, 9.21)	<0.001	6.23 (3.52,13.22)	<0.001
	Q4	5.06 (3.27, 11.64)	<0.001	7.96 (5.38,16.27)	<0.001
**BMI (kg/m^2^)**					
**<25**					
	Q1	Reference		Reference	
	Q2	2.30 (0.78, 6.73)	0.128	5.19 (2.19,12.61)	0.028
	Q3	3.26 (1.17, 7.05)	<0.001	7.72 (4.72,13.49)	<0.001
	Q4	5.49 (2.03, 9.79)	<0.001	9.94 (6.68,20.75)	<0.001
**≥25**					
	Q1	Reference		Reference	
	Q2	6.65 (1.47, 13.05)	0.014	2.37 (0.82,6.85)	0.108
	Q3	7.89 (4.12, 17.58)	<0.001	3.14 (1.14,8.66)	0.027
	Q4	9.32 (3.83, 19.57)	<0.001	9.93 (3.72,26.49)	<0.001
**Hypertension**					
**No**					
	Q1	Reference		Reference	
	Q2	4.45 (1.83, 7.15)	0.011	2.36 (0.62,8.91)	0.203
	Q3	7.28 (6.31, 14.04)	<0.001	5.02 (2.32,9.75)	0.001
	Q4	8.42 (7.63, 17.12)	<0.001	7.08 (4.23,18.21)	<0.001
**Yes**					
	Q1	Reference		Reference	
	Q2	1.45 (0.54, 3.86)	0.457	2.91 (0.99,8.58)	0.052
	Q3	7.60 (3.15, 18.32)	<0.001	6.088 (2.14,17.27)	0.001
	Q4	9.26 (3.01, 17.50)	<0.001	7.963 (2.79,22.72)	<0.001
**Diabetes**					
**No**					
	Q1	Reference		Reference	
	Q2	4.59 (1.28, 7.46)	0.019	3.91 (1.33,7.51)	0.013
	Q3	6.40 (3.90, 13.78)	<0.001	4.33 (3.56,8.96)	<0.001
	Q4	6.80 (5.99, 13.40)	<0.001	5.94 (3.53,9.99)	<0.001
**Yes**					
	Q1	Reference		Reference	
	Q2	2.58 (0.81, 8.24)	0.109	1.62 (0.43,6.11)	0.475
	Q3	4.34 (3.84, 7.44)	<0.001	3.92 (1.18,7.05)	0.026
	Q4	9.77 (3.75, 18.89)	<0.001	7.06 (2.74,18.91)	<0.001
**AKI**					
**No**					
	Q1	Reference		Reference	
	Q2	3.93 (1.08, 5.29)	0.037	3.92 (0.87,6.53)	0.073
	Q3	5.43 (8.02, 8.05)	<0.001	6.13 (3.78,10.72)	<0.001
	Q4	6.74 (6.59, 11.64)	<0.001	4.09 (7.44,9.95)	<0.001
**Yes**					
	Q1	Reference		Reference	
	Q2	3.19 (1.01, 7.05)	0.047	3.10 (1.12,8.55)	0.028
	Q3	4.26 (2.48, 9.25)	<0.001	5.52 (1.73,11.85)	0.002
	Q4	5.43 (3.64, 9.89)	<0.001	4.44 (3.14,9.66)	<0.001

## 4 Discussion

This study investigated the relationship between insulin resistance and 28-day mortality in patients undergoing VA-ECMO therapy. Insulin resistance is a pathological condition characterized by a reduced sensitivity of target tissues to insulin and impaired glucose uptake and metabolism, contributing to metabolic dysregulation. This disorder plays a crucial role in the pathogenesis of various metabolic disorders, including type 2 diabetes mellitus and cardiovascular diseases, both of which are associated with significant morbidity and mortality. This condition is characterized by impaired insulin action, leading to metabolic disturbances such as dyslipidemia, which can negatively influence the prognosis of patients with acute illnesses requiring intensive care, including those on VA-ECMO support. The significance of insulin resistance, especially in the context of metabolic syndrome, has garnered considerable attention due to its association with adverse clinical outcomes, including increased mortality among critically ill patients (15, 16). Changes in lipid profiles, particularly in TG and HDL cholesterol levels, are essential biomarkers for assessing the severity of insulin resistance and may correlate with heightened mortality risk in critically ill patients (8, 17, 18).

This retrospective study evaluated the association between multiple insulin resistance indices (such as the TyG index, METS-IR, TG/HDL-C ratio, and TyG-BMI index) and 28-day mortality in patients on VA-ECMO. Our findings indicate that elevated insulin resistance indices are significantly associated with higher mortality rates, underscoring the importance of monitoring and managing insulin resistance to improve outcomes in patients receiving VA-ECMO support. Insulin resistance indicators (TyG, TG/HDL-C) can independently and gradually predict the risk of death in patients with VA-ECMO. The model combined with indicators such as SOFA score has high discriminative power and clinical practicability. This provides new evidence for risk stratification based on the integration of metabolism and organ function, supporting the research exploration of targeted intervention for insulin resistance to improve prognosis.

TyG, METS-IR, TG/HDL-C, and TyG-BMI independently predicted an increased risk of death (all *p* < 0.01). Five core predictors were determined through multi-method screening: Sequential Organ Failure Assessment (SOFA) score, TyG, TG/HDL-C, hypertension, and diabetes. The joint model performed excellently in both the training set and the validation set. [Training set: area under curve (AUC) = 0.923, C index = 0.847, NRI = 0.699, IDI = 0.175; Validation set: AUC = 0.901, C index = 0.846, NRI = 0.574, IDI = 0.148], and the DCA and calibration curve show its good efficacy. The association between insulin resistance and clinical outcomes in patients on VA-ECMO has garnered considerable attention. The TG/HDL-C ratio, widely recognized as a marker of atherosclerotic dyslipidemia, is strongly associated with cardiovascular risk and mortality (19). Our findings confirm this, showing that higher TG/HDL-C ratios are associated with worse 28-day survival in patients on VA-ECMO. Recent studies have suggested that the TG/HDL-C ratio may serve as a reliable biomarker for predicting mortality in patients with conditions such as chronic kidney disease and non-small-cell lung cancer (20, 21). The level of TG/HDL-C in patients with coronary artery disease is significantly increased. HDL-C not only has the reverse cholesterol transport function, but also can protect blood vessels through antioxidant and anti-inflammatory effects (22). The high TG/HDL-C level in patients with hypertension requires closer blood pressure monitoring, as it may indicate poor blood pressure control in this population (23). Studies have shown that the level of TG/HDL-C is significantly positively correlated with glycated hemoglobin and blood glucose levels before eating and drinking. Therefore, TG/HDL-C in patients with type 2 diabetes should be monitored in order to detect diabetic microvascular complications in a timely manner (24). Excess dietary fat is stored in adipose tissue as TG. When TG accumulation exceeds a critical threshold, it disrupts glucose metabolism and inhibits insulin action on receptors in peripheral tissues, resulting in insulin resistance. Conversely, reduced HDL-C levels lead to excessive cholesterol buildup in insulin-sensitive tissues, triggering insulin resistance through inflammation and macrophage infiltration (25). This evidence collectively underscores the utility of TG/HDL-C as a valuable biomarker in critically ill populations. The SOFA score is a widely recognized scale for assessing disease severity, particularly sepsis. It is extensively used as a reference index in infection-related studies, as it dynamically reflects changes in organ function in patients with multiple organ failure (26). Infection-related complications frequently occur during VA-ECMO therapy, following a continuum from infection to sepsis, progressing to organ dysfunction, and eventually culminating in multiple organ failure. Therefore, dynamic monitoring of the SOFA score is crucial for evaluating patient status. The SOFA score effectively predicts outcomes in critically ill patients receiving VA-ECMO support (27). Incorporating markers such as the SOFA score, TG/HDL-C ratio, and other related indices into clinical practice can aid in identifying high-risk patients who could benefit from more aggressive and targeted management strategies.

The TyG index, calculated as the natural logarithm of the product of fasting TG and glucose levels, has gained recognition as a reliable surrogate marker for insulin resistance. Previous studies (28) have highlighted its strong association with cardiovascular events and metabolic disorders, supporting its relevance in predicting outcomes in critically ill patients, including those receiving VA-ECMO support. Similarly, the inclusion of BMI in evaluating insulin resistance through the METS-IR index reinforces the critical role of metabolic dysfunction in determining outcomes in the intensive care unit (29). The TyG-BMI index emerged as a robust predictor of mortality, suggesting the importance of overall body composition, along with glucose and TG levels, in assessing the risk of death in this patient cohort (8). Identifying specific cutoff values for these indicators can enhance predictive accuracy and enable more personalized treatment planning.

Our findings highlight the significance of markers of insulin resistance as predictors of 28-day mortality in patients with VA-ECMO. Incorporating indices such as the TyG index, METS-IR, TG/HDL-C ratio, and TyG-BMI in routine clinical evaluations can facilitate the early identification of high-risk patients, improving management strategies and survival outcomes for this vulnerable population. Future studies should focus on validating these measures in larger cohorts and exploring their potential role in guiding therapeutic interventions.

Studies have found that insulin resistance is associated with high insulin demand, metabolic disorders and increased mortality. Among critically ill patients, the mechanism of insulin resistance is complex, involving multiple physiological and pathological processes, including inflammatory responses, the secretion of stress hormones, and metabolic disorders, etc. (30). The insulin signaling pathway plays a key role in maintaining glucose metabolism and energy balance. Insulin binds to insulin receptors and activates downstream signal transduction pathways, including the PI3K/Akt pathway and the MAPK pathway, which are crucial in regulating glucose transport and fat synthesis. However, in critically ill patients, abnormalities in the insulin signaling pathway often lead to the occurrence of insulin resistance. Studies have shown that the phosphorylation level of insulin receptors in critically ill patients is decreased, resulting in impaired insulin signal transduction and subsequently affecting the uptake and utilization of glucose (31). In addition, the increase of inflammatory factors and stress hormones can also interfere with the insulin signaling pathway, further aggravating the degree of insulin resistance (32). Hyperglycemia is a common metabolic disorder in critically ill patients and is usually closely related to insulin resistance. Studies show that persistent high blood sugar can cause damage to multiple organs, especially the heart, kidneys and nervous system. Under hyperglycemic conditions, the metabolic function of the heart is inhibited, leading to abnormal energy metabolism in myocardial cells and increasing the risk of heart disease (33). Insulin resistance is a decrease in the body’s sensitivity to insulin, leading to compensatory hyperinsulinemia, which is the core pathophysiological mechanism of various metabolic diseases. In the early stage of insulin resistance, pancreatic islet β cells maintain blood glucose homeostasis by secreting more insulin (i.e., the “compensatory phase”). However, long-term hyperinsulinemia can aggravate insulin resistance and form a vicious cycle.

This study has several limitations that should be considered. First, as a retrospective analysis, it is inherently prone to selection bias and confounding factors, which may influence the association between measures of insulin resistance and 28-day mortality in patients on VA-ECMO. The reliance on historical medical records for baseline disease identification introduces the potential for inaccuracies, as data quality depends on the precision and completeness of the initial documentation. Moreover, the study did not consider potential therapeutic interventions or changes in clinical management after VA-ECMO initiation, which could have significantly influenced mortality outcomes. Finally, while multiple measures of insulin resistance were evaluated, the interactions between these indices and other unmeasured variables remain unclear, potentially limiting a comprehensive understanding of their prognostic value.

## 5 Conclusion

This study identified measures of insulin resistance, specifically the TyG index, METS-IR, TG/HDL-C ratio, and TyG-BMI index, as significant predictors of 28-day mortality in patients with VA-ECMO. These findings underscore the importance of incorporating metabolic parameters into prognostic assessments for critically ill patients receiving VA-ECMO support. Future prospective studies are warranted to validate these associations and explore potential mechanisms linking insulin resistance to poor outcomes in this population. A deeper understanding of these relationships may enable the development of tailored treatment interventions and enhanced patient management strategies in VA-ECMO therapy.

## Data Availability

The raw data supporting the conclusions of this article will be made available by the authors, without undue reservation.
