# Supplementary figures and images for: Indicators of insulin resistance as predictors of 28-day mortality in patients with VA-ECMO: a retrospective study
Source: Front Med (Lausanne). 2025 May 22;12:1559780. doi: 10.3389/fmed.2025.1559780 (PMC12137338; doi:10.3389/fmed.2025.1559780)

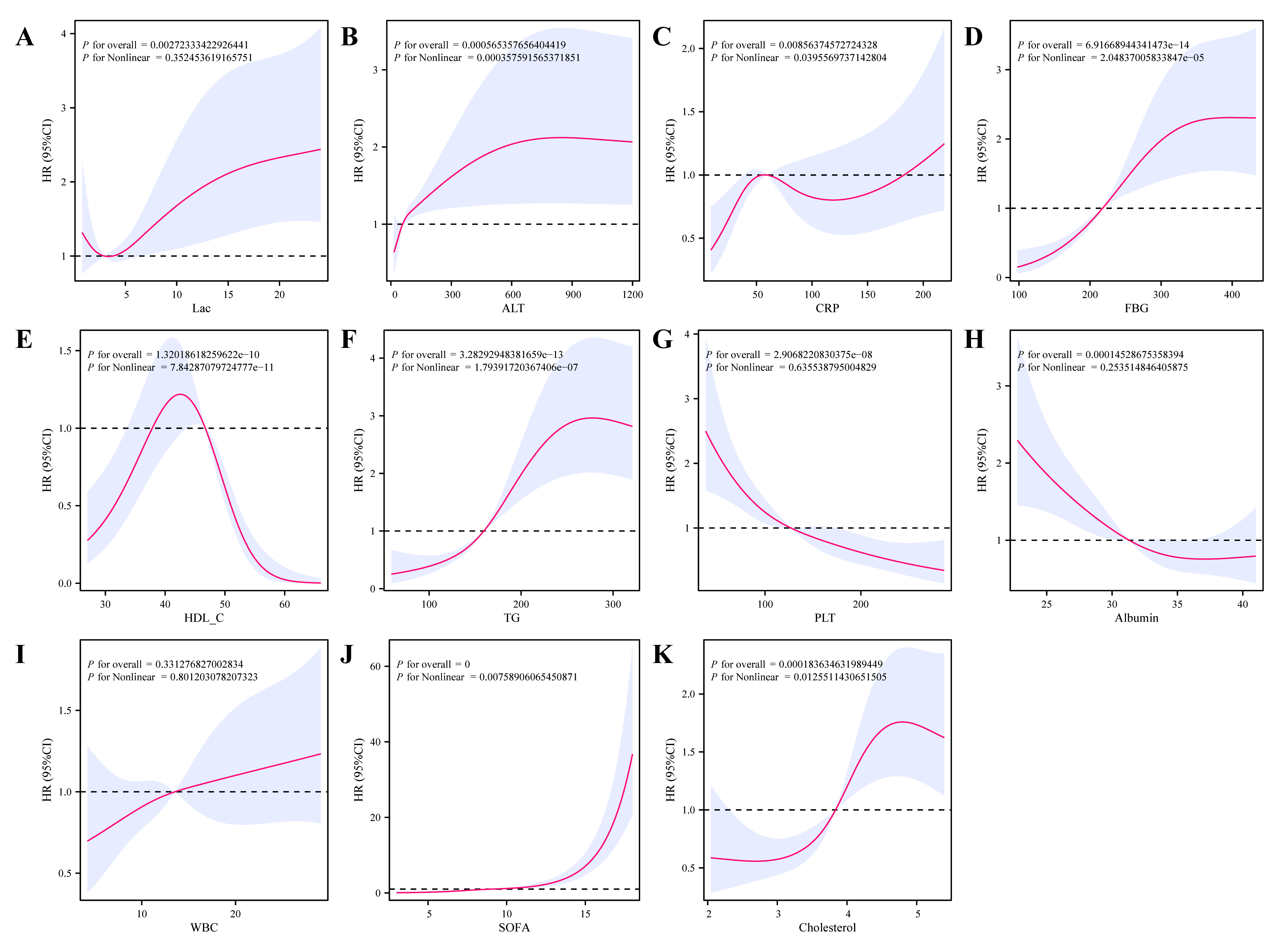

Supplement: Supplementary file 1 [file Image_1.tif]
