# Supplementary material for: Indicators of insulin resistance as predictors of 28-day mortality in patients with VA-ECMO: a retrospective study
Source: Front Med (Lausanne). 2025 May 22;12:1559780. doi: 10.3389/fmed.2025.1559780 (PMC12137338; doi:10.3389/fmed.2025.1559780)
Supplement: Supplementary file 2 [file Table_1.docx]

Table S1: The variance inflation factor (VIF) values of TyG, METS_IR, TG/HDL_C, and TyG_BMI in the multifactor Cox regression.

| Variable | TyG | METS_IR | TG/HDL_C | TyG_BMI |
| --- | --- | --- | --- | --- |
| Sex | 1.229 | 1.215 | 1.244 | 1.254 |
| Hypertension | 1.155 | 1.173 | 1.159 | 1.201 |
| Diabetes | 1.217 | 1.169 | 1.234 | 1.162 |
| AKI | 1.415 | 1.386 | 1.406 | 1.389 |
| Age | 1.254 | 1.261 | 1.233 | 1.228 |
| BMI | 1.463 | - | 1.439 | - |
| WC | 1.601 | 1.582 | 1.582 | 1.531 |
| SOFA | 1.711 | 1.502 | 1.693 | 1.526 |
| APACHE_II | 1.515 | 1.397 | 1.397 | 1.359 |
| WBC | 1.083 | 1.102 | 1.092 | 1.097 |
| PLT | 1.214 | 1.200 | 1.235 | 1.200 |
| AST | 1.153 | 1.143 | 1.151 | 1.150 |
| Albumin | 1.109 | 1.103 | 1.123 | 1.099 |
| CR | 1.488 | 1.477 | 1.479 | 1.505 |
| BUN | 1.483 | 1.428 | 1.422 | 1.438 |
| Cholesterol | 1.290 | 1.232 | 1.265 | 1.233 |
| Lac | 1.204 | 1.196 | 1.198 | 1.203 |
| TG | - | - | - | - |
| HDL_C | 1.362 | - | - | 1.23 |
| IL_6 | 1.140 | 1.146 | 1.147 | 1.139 |
| CRP | 1.121 | 1.125 | 1.121 | 1.115 |
| FBG | - | - | 1.503 | - |
| TyG1 | 2.122 | - | - | - |
| METS_IR1 | - | 1.969 | - | - |
| TGHDL_C1 | - | - | 1.632 | - |
| TyG_BMI1 | - | - | - | 1.811 |
